# Mitochondrial DNA disease and developmental implications for reproductive strategies

**DOI:** 10.1093/molehr/gau090

**Published:** 2014-11-24

**Authors:** Joerg Patrick Burgstaller, Iain G. Johnston, Joanna Poulton

**Affiliations:** 1 Biotechnology in Animal Production, Department for Agrobiotechnology, IFA Tulln, 3430 Tulln, Austria; 2 Institute of Animal Breeding and Genetics, University of Veterinary Medicine Vienna, Veterinärplatz 1, 1210 Vienna, Austria; 3 Department of Mathematics, Imperial College London, London SW7 2AZ, UK; 4 Nuffield Department of Obstetrics and Gynaecology, University of Oxford, Oxford OX3 9DU, UK

**Keywords:** mitochondrial DNA, haplotype matching, mtDNA segregation, development, preventing mtDNA disease

## Abstract

Mitochondrial diseases are potentially severe, incurable diseases resulting from dysfunctional mitochondria. Several important mitochondrial diseases are caused by mutations in mitochondrial DNA (mtDNA), the genetic material contained within mitochondria, which is maternally inherited. Classical and modern therapeutic approaches exist to address the inheritance of mtDNA disease, but are potentially complicated by the fact that cellular mtDNA populations evolve according to poorly-understood dynamics during development and organismal lifetimes. We review these therapeutic approaches and models of mtDNA dynamics during development, and discuss the implications of recent results from these models for modern mtDNA therapies. We particularly highlight mtDNA segregation—differences in proliferative rates between different mtDNA haplotypes—as a potential and underexplored issue in such therapies. However, straightforward strategies exist to combat this and other potential therapeutic problems. In particular, we describe haplotype matching as an approach with the power to potentially ameliorate any expected issues from mtDNA incompatibility.

## Introduction: mitochondrial DNA disease

Mitochondria are vital energy-producing organelles in eukaryotic cells. Mitochondrial diseases are pathologies in which the ability of mitochondria to produce energy and fulfill their normal cellular roles is compromised. These diseases are relatively common, but diagnosed infrequently, because the majority of patients exhibit only very mild symptoms (Manwaring *et al.*, 2007). The range of symptomatic severity associated with mitochondrial disease leads to variability in reported prevalence rates: for example, one mitochondrial pathology (resulting from the m.3243A>G mutation discussed below) has quoted prevalence rates of between 1 in 300 (Manwaring *et al.*, 2007) and 1 in 14 000 (Chinnery *et al.*, 2000).

Mitochondria have their own DNA (henceforth mtDNA), which is the only DNA outside the nucleus in humans. MtDNA is unique in having its own genetic code and subtype of ribosomes. It codes for a minority of subunits required for the respiratory chain, the array of multimeric proteins that form the production line for energy on the inner mitochondrial membrane, and for transfer and ribosomal RNA. The majority of essential respiratory chain proteins are encoded by the nucleus, as well as many proteins required for mtDNA maintenance and replication. Therefore, mutations in either the mitochondrial or nuclear DNA of the cell may cause pathological loss of function in mitochondria and lead to mitochondrial disease (Taylor and Turnbull, 2005; Greaves *et al.*, 2012). In this review, we will focus on diseases resulting from mtDNA mutation. MtDNA is maternally inherited, apparently because sperm contribute almost no cytoplasm to the zygote, and paternal mitochondria are ubiquitinated (Sutovsky *et al.*, 1999, 2000) and targeted for destruction (Cummins *et al.*, 1998; Shitara *et al.*, 2000; Al Rawi *et al.*, 2011; Sato and Sato, 2011; DeLuca and O'Farrell, 2012) as soon as fertilization has taken place, persisting only in abnormal embryos or inter-species crosses (Gyllensten *et al.*, 1991; St John *et al.*, 2000). MtDNA is possibly even eliminated prior to fertilization (Luo *et al.*, 2013).

Diseases resulting from mutations in mtDNA have unique characteristics of onset, severity and inheritance, largely due to the fact that there are thousands of copies of mtDNA in a typical nucleated cell (Lightowlers *et al.*, 1997; Wallace, 1999). In most normal individuals these are effectively genetically identical (a situation termed ‘homoplasmy’). In mtDNA disease there may be a number of different, mutated mtDNA molecules, giving rise to ‘heteroplasmy’ (more than one mtDNA type coexisting in the same cell).

MtDNA is maternally inherited, producing a characteristic disease distribution down the maternal line. MtDNA haplotypes can modulate the pathological effects of mutated nuclear encoded genes (Strauss *et al.*, 2013), and the same mtDNA variation can be deleterious or beneficial depending on its mtDNA background and environment (Ji *et al.*, 2012). Many mtDNA diseases are heteroplasmic, that is, both mutated and wild-type mtDNA co-exist in affected individuals. In most of these cases a dosage effect is observed (Jeppesen *et al.*, 2006), with the proportion, copy number and distribution of mtDNA mutants influencing tissue function (Petruzzella *et al.*, 1994). The ‘threshold’ above which mtDNA disease shows clinical symptoms is around 70% mutated mtDNA in the commonest disorder (Jeppesen *et al.*, 2006). This dependence on mutant load is important because intracellular populations of mtDNA, and thus the proportional presence of mutant mtDNA, can change during development, according to dynamics which are currently poorly characterized.

The developmental modulation of mtDNA populations means that patients with mtDNA disease frequently exhibit progressive symptoms, as mutant mtDNA accumulates in affected tissues (Poulton *et al.*, 1995; Weber *et al.*, 1997). For instance, children with Pearson's syndrome may present with severe anemia and lactic acidosis in early infancy. The characteristic mutation is a single mtDNA deletion of about 5 kilobases, encompassing both protein and tRNA coding regions. Affected children initially have high levels of mutant mtDNA in all tissues. As the disease progresses, the level of mutant in blood drops and their anemia resolves. However, if they survive to adolescence they may develop a myopathy as the proportion of mutant mtDNA in muscle increases (McShane *et al.*, 1991). While the shifting of mtDNA populations is less extreme in most maternally inherited heteroplasmic mtDNA disease, in almost all cases the level of mutant mtDNA is lower in blood than in post-mitotic tissues such as muscle and brain (Rahman *et al.*, 2001). This example illustrates a potential diagnostic problem: as mutant load changes with time, blood levels of mutant mtDNA cannot easily be used to advise patients on their prognosis or transmission risks.

Mitochondrial diseases are often clinically heterogeneous. While many patients do not fit into specific clinical syndromes, well known examples of mtDNA diseases include MIDD (mitochondrially inherited diabetes and deafness) (van den Ouweland *et al.*, 1992), MELAS (mitochondrial myopathy, encephalomyopathy, lactic acidosis, stroke-like symptoms) (Goto *et al.*, 1990), MILS (maternally inherited Leigh's syndrome) (Holt *et al.*, 1990), MERRF (myoclonic epilepsy with ragged red fibers) (Wallace *et al.*, 1988b) and LHON (Leber's hereditary optic neuropathy) (Wallace *et al.*, 1988a; Howell and McCullough, 1990; Johns *et al.*, 1992). Muscle dysfunction is an important feature of MELAS (Ciafaloni *et al.*, 1992) and MERRF, both of which can cause cognitive decline, ataxia, epilepsy, cardiomyopathy and deafness (Chinnery *et al.*, 1997). Diabetes is a common feature of MELAS (van den Ouweland *et al.*, 1992). MILS mainly involves the central nervous system with psychomotor delay, visual and hearing impairment (Degoul *et al.*, 1995). LHON is usually a non-syndromic optic neuropathy and most patients are homoplasmic for mutant mtDNA (Howell and McCullough, 1990; Johns *et al.*, 1992). Specific mutations in mtDNA are known to give rise to these diseases: for example, the m.3243A>G mutation most often causes MIDD, but in more severe cases MELAS (Goto *et al.*, 1990), the m.8344A>G mutation can cause MERRF (Wallace *et al.*, 1988b), and m.11778G>A (Wallace *et al.*, 1988a), m.3460G>A (Howell and McCullough, 1990) and m.14484T>C (Johns *et al.*, 1992) mutations can cause LHON. However other mtDNA mutations also give rise to these diseases. A review of clinical features and a morbidity map of mtDNA mutations can be found in Chinnery and Hudson (2013) and DiMauro *et al.* (2013).

**Figure 1:**
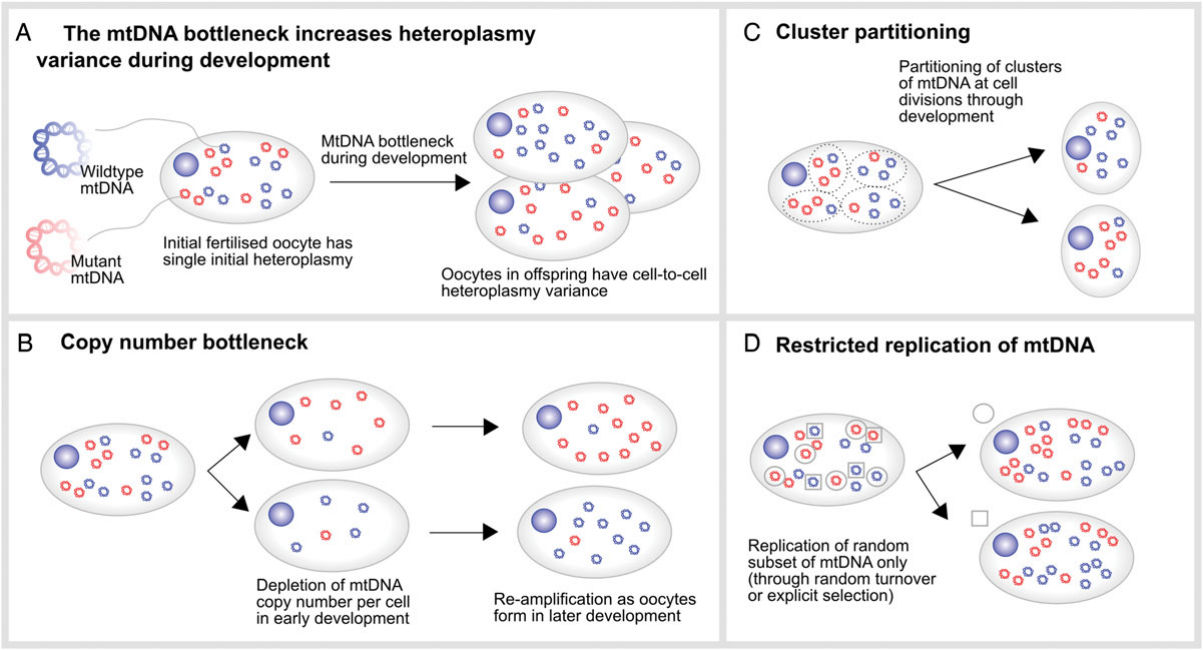
The mitochondrial DNA bottleneck during development. (**A**) A fertilized oocyte has a given heteroplasmy (mutant load) value. During gestation, the female embryo/fetus develops primordial germ cells that develop into oocytes. The heteroplasmy in these oocytes shows high variance due to the bottleneck effect, whose proposed mechanisms are shown in (**B**)–(**D**). (B) A reduction of mitochondrial DNA (mtDNA) copy number in the primordial germ cells and consecutive reamplification during oocyte development accelerates random drift and increases variance. (C) Random partitioning of clusters of mtDNAs at each cell division during primordial germ cell development could powerfully increase heteroplasmy variance. (D) Allowing only a small random subset of mtDNAs to replicate (here two instances are depicted with circles and squares)—either a specifically selected set or through restricted random turnover—can increase heteroplasmy variance through imposing a lower effective population size.

Another striking feature of mtDNA disease inheritance involves the observed large shifts of heteroplasmy between mother and offspring. For example, it is possible for a phenotypically healthy mother, harboring 50% mutated mtDNA, to produce both healthy and severely affected children (Larsson *et al.*, 1992). The reason for this shift between generations is the so-called ‘bottleneck’ effect, whereby heteroplasmy levels in offspring are remarkably variable with respect to the maternal heteroplasmy, while the average heteroplasmy across many offspring is often comparable to that of the mother (Jenuth *et al.*, 1996). The mechanism underlying this effect is hotly debated (Carling *et al.*, 2011), with some suggesting that responsibility lies with a pronounced decrease in mtDNA copy number in the germline (Cree *et al.*, 2008), others proposing random partitioning of clusters of mtDNA at cell divisions (Cao *et al.*, 2007, 2009), and others proposing replication of a subset of mtDNAs during development (Wai *et al.*, 2008) (Fig. 1).

In the most extreme examples of the bottleneck, there may be rapid switching from near homoplasmy in one mtDNA type to near homoplasmy for another between mother and child. Using a heteroplasmic length variant in noncoding mtDNA (Marchington *et al.*, 1997) one of the current authors (J.P.) found evidence for this switching in oocytes from women and from mice. This switching was also apparent in oocytes from women who were heteroplasmic for pathogenic mtDNA mutants (Blok *et al.*, 1997; Marchington *et al.*, 1998; Brown *et al.*, 2001). While further work is needed to elucidate this mechanism (Carling *et al.*, 2011), the bottleneck is a clear example of how developmental phenomena mold the statistics of intracellular mtDNA populations.

**Figure 2:**
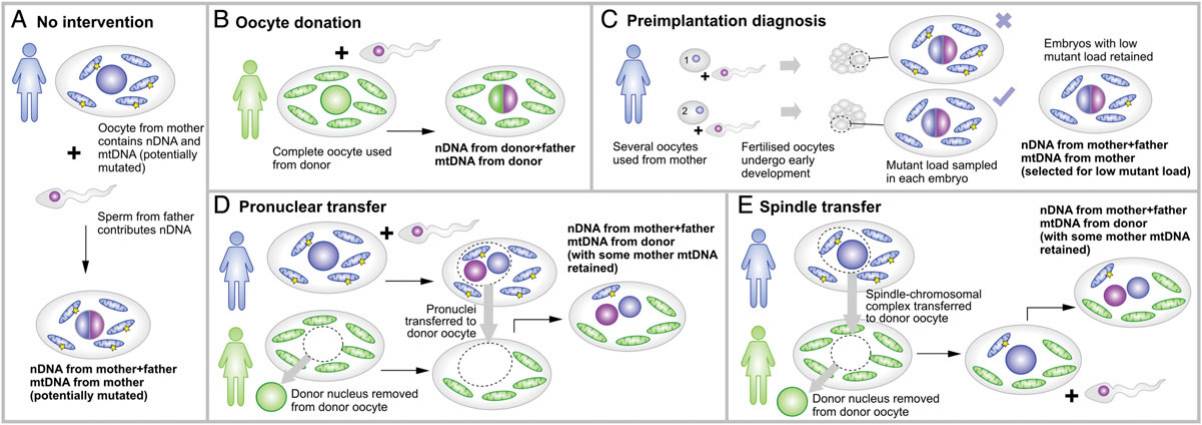
MtDNA disease inheritance and therapeutic approaches. (**A**) A mother with mtDNA harboring a pathological mutation is at risk of transmitting the associated disease to her offspring. (**B**) Oocyte donation uses an oocyte from a third-party donor who does not carry the mtDNA mutation. (**C**) Preimplantation diagnosis involves sampling mutant load in cells after conception. As the mother's oocytes may exhibit a wide range of heteroplasmy levels, some concepti may inherit acceptably low mutant loads: these are retained. (**D**) Pronuclear transfer involves the removal of the nucleus from a third-party oocyte with unaffected mtDNA, then the transfer of two pronuclei (from the mother's egg and father's sperm) onto this healthy background. (**E**) Spindle transfer involves the replacement of a third-party oocyte nucleus with the chromosomal complex from the mother, prior to fertilization with the father's sperm.

MtDNA diseases are currently not directly curable, despite several promising approaches to shift the amount of mutated mtDNA in patients affected by heteroplasmic diseases to lower, less pathogenic levels. For example, specifically designed nucleases (including so-called mitoTALENs (Bacman *et al.*, 2013) and zinc-finger nucleases (Gammage *et al.*, 2014)) can cut mutated mtDNA at the site of the mutation. In the absence of clinically available cures of mtDNAs disease, strategies to prevent their transmission to the next generation to allow (subclinically) affected women to have healthy children (or at least highly increase the chances thereof) are extremely important (Fig. 2). We designate those therapeutic strategies that are in current clinical practice as ‘Classical’ and those that have not yet been approved for use in humans as ‘Modern’. Classical strategies aim to either replace the affected oocyte from the patient altogether, or monitor embryo/fetal heteroplasmy. Modern strategies aim at replacing the affected mtDNA. These new strategies have become prominent in the scientific literature and media alike. The appealing catchphrases ‘like changing a laptop battery’ (referring to the replacement of dysfunctional mtDNA) and associated ‘three-parent babies’ (referring to the presence of a third-party's mtDNA in an embryo; see below) have captured the imagination of many involved in communicating science to the general public, and several of these recently proposed therapies for mtDNA disease inheritance are currently on the verge of clinical application.

## Classical options in reproductive management of mtDNA disease

Three notable strategies have existed, at least in concept (Sauer and Kavic, 2006), since before the first maternally inherited mtDNA disease was described 25 years ago (Wallace *et al.*, 1988b), to address the issue of potential inheritance of mutant mtDNA in families at risk of transmitting diseases (Fig. 2A).

Oocyte donation (Fig. 2B) is a simple way to completely eliminate the risk of transmitting heteroplasmic mutant mtDNA from mother to child. This approach involves using the oocytes from a third-party donor rather than the mother, thus losing any genetic inheritance from the mother, but representing the only strategy guaranteeing to intercept the transmission of the disease.

Alternative strategies exploit the difference in mutant load between a potential mother's oocytes, arising largely from the aforementioned mtDNA bottleneck, and selected concepti with a low mutant load. This strategy can only be applied in heteroplasmic disease. Selection of low risk concepti was initially carried out during established pregnancy (chorionic villus sampling) (Harding *et al.*, 1992). Here, heteroplasmy in chorionic villi is analyzed at the end of the first trimester, with a view to terminating fetuses displaying high heteroplasmies and thus at high risk of inheriting mtDNA disease. This approach is useful to address mtDNA disease inheritance in disorders where there is a good relationship between phenotype and mutant load (White *et al.*, 1999). It is not suitable for homoplasmic diseases, nor for those in which the mtDNA mutant load is a poor predictor of phenotype, like LHON (Black *et al.*, 1996). Furthermore, if the load of mutant mtDNA in trophoblast is a poor representation of that in the rest of the conceptus, the efficacy of this approach is decreased. This poor representation can be the case when segregation starts early, as described in the next paragraph.

A third therapeutic strategy involves selecting low risk early (‘cleavage’) embryos, through the use of preimplantation genetic diagnosis (PGD) ((Fig. 2C). After fertilization of an oocyte and limited subsequent development, a small fraction (1–2 cells) of the cleavage embryo is sampled to determine the mutant load. At this stage, the variation in mutant load between individual blastomeres is small. The mutant load in an embryo can then be used to estimate the risk of the individual developing symptoms of a mtDNA disorder post-natally (Poulton *et al.*, 2010). This strategy is currently the mainstay of modern clinical practice, being used successfully to help families with mtDNA disease (Steffann *et al.*, 2006; Monnot *et al.*, 2011). However, when PGD is carried out on blastocysts, a later embryonic stage, samples may not adequately reflect post-natal heteroplasmy. This problem arose when a variant of the technique, blastocyst biopsy, was used for prenatal screening of an embryo carrying the m.3243A>G mutation (Treff *et al.*, 2012). In this instance, the mutant load in trophoblast cells (12%; Treff *et al.* (2012)) was substantially lower than in some samples of the child (47% blood, 52% urine; Wallace and Chalkia (2013) and Mitalipov *et al.* (2014)). It is currently unknown whether the difference in heteroplasmy occurred between trophoblast and inner cell mass, or whether the heteroplasmy levels changed during gestation. However, this case shows the considerable residual risk of this method. Generally, cell-to-cell heteroplasmy and copy number variation are likely to develop as cells develop down the specific functional lineages found in the blastocyst. Such variation could be further exacerbated by a proposed rapid mtDNA segregation in preimplantation embryos (Lee *et al.*, 2012). However, it needs to be clarified whether this is a general phenomenon, or a result of the merging of two distinct cytoplasts that segregate independently in ‘artificially generated embryos’. If the latter is true, the effect will be of importance in all techniques that include some degree of cytoplasmic transfer, including karyoplast transfer (Steffann *et al.*, 2014). In conclusion, PGD on cleavage stage embryos seems robust, but on blastocysts may be unreliable.

## New developments: modern treatments for mtDNA disease

The above approaches to address the inheritance of mitochondrial disease have several shortcomings. Oocyte donation has the disadvantage that none of the mother's nuclear DNA content is retained in the offspring. PGD of blastocysts (but not of cleavage stage embryos) and chorionic villus sampling run the risk that differences between tissues and individual cells may lead to an inaccurate inference of heteroplasmy levels, and thus erroneous risk estimation. PGD of cleavage stage embryos however appears to be robust and accurate (Monnot *et al.*, 2011).

Two recently proposed therapies, pronuclear transfer and chromosomal spindle transfer, are designed to circumvent these problems. Both these approaches aim to transfer the nuclear genome of a parent ‘donor’ oocyte to a healthy ‘recipient’ oocyte with no nucleus and healthy mtDNA. Specifically, pronuclear transfer (Fig. 2D) involves transferring the two pronuclei (from mother and father) of a fertilized donor oocyte to an enucleated recipient oocyte. Chromosomal spindle transfer (Fig. 2E) involves transferring the chromosomal spindle from a donor oocyte into an enucleated recipient oocyte prior to fertilization. Thus, the nuclear genome is transferred to an environment with functional mitochondria, i.e. the defective ‘batteries’ of the cells are replaced with working ones; resulting in a healthy embryo and definitely interrupting inheritance of the pathological mtDNA (Poulton and Oakeshott, 2012). These therapies aim to achieve zero mutant load, consistent across all cells, and allow the mother and father both to contribute nuclear DNA, thus addressing the shortcomings of traditional therapies.

Pilot studies of these techniques have proved their feasibility, but also highlighted the potentially important and currently unavoidable phenomenon of mtDNA carryover. Ideally, pronuclear and spindle transfer both should lead to a complete lack of donor mtDNA in the recipient, but technical limitations currently make this unfeasible (St John and Campbell, 2010). Currently, no method has been shown to reproducibly eliminate 100% of the unwanted donor mtDNA carryover. While all techniques tend to reduce carryover to below 1%, comparison between the studies is hampered by varying detection limits of donor mtDNA, between 0.01 and 2%. In five out of nine human embryos created by pronuclear transfer (Craven *et al.*, 2010) the average carryover of donor mtDNA was 1.68%. Observed carryovers in other studies include 0.5–0.6% from human spindle transfer (Tachibana *et al.*, 2013); 0.31% in human nuclear transfer (Paull *et al.*, 2013); and 1% in rhesus monkeys with spindle transfer (Tachibana *et al.*, 2009; Lee *et al.*, 2012). Therefore, a low-level heteroplasmy in the resulting embryo cannot be excluded.

Such low amounts are generally insufficient to cause disease (Craven *et al.*, 2011). The mtDNA bottleneck may conceivably lead to amplification in the next generation of this amount in female embryos: however, it was recently shown that for carryover <5%, this is no concern also for the following generations, and both methods should therefore be safe in this respect (Samuels *et al.*, 2013).

## Potential issues with modern treatments

While these modern treatments are largely deemed sufficiently safe for use in the clinic, several uncertainties exist regarding the behavior of mtDNA populations after treatment. Until these uncertainties are addressed, clinical applications of modern treatments should arguably be limited to cases where no good alternatives exist. Families with severe phenotypes and a homoplasmic mtDNA mutant of proven pathogenicity are the best candidates, because the only classical option from which they benefit is oocyte donation. However, such families are relatively rare, largely because it is essential but difficult to prove that a homoplasmic mutation is causative. Correct selection of the first families for treatment will therefore be imperative.

At the current time most possible and ethically justifiable pilot tests have been performed both in animal models and (abnormal) human embryos, with positive results. However, some questions regarding the subsequent behavior of mtDNA populations in offspring produced using these treatments remain, which have been flagged by researchers and noted in the literature. These issues are not fatal flaws in the concept of mtDNA therapies, but do represent areas of uncertainty associated with these therapies. All of them are connected with the mtDNA haplotype of the third-party ‘recipient’ providing an oocyte with healthy mtDNA. In a random pairing, it is likely that the donor and recipient haplotypes vary considerably: pairwise comparisons in human mtDNAs show up to 130 single-nucleotide polymorphism (SNP) differences ((Blanco *et al.*, 2011), with those located in the protein-coding regions of the mtDNA leading to up to 20 amino acid changes (Craven *et al.*, 2011)). On average two Europeans or two Africans will differ at 29.3 and 78.3 sites, respectively (Lippold *et al.*, 2014). Consequently, a rather complex mixture of nuclear DNA and different mtDNAs can arise: nuclear DNA from the patient (‘donor’) and the father, the majority of mtDNA from the enucleated recipient oocyte with presumed wild-type mtDNA (haplotype B) and a small amount of the carryover patient mtDNA (haplotype A; which may be either mutant or wild type if the donor is heteroplasmic). So a maximum of three different mtDNAs can be present in the embryo, with the healthy haplotype B constituting the vast majority, around 99%. This new haplotype is alien to both the patient (maternal) and paternal nuclear DNA, and the implications of this combination are currently largely unexplored.

**Figure 3:**
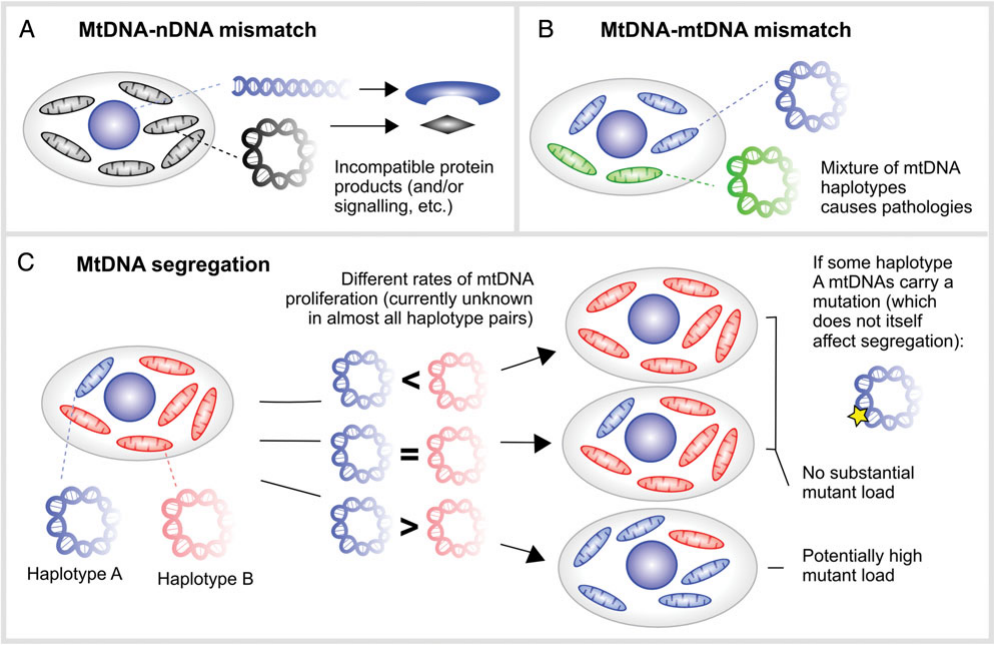
Potential issues associated with mixed mtDNA populations resulting from modern therapies. (**A**) Incompatibilities may exist between the nuclear DNA (from mother and father) and mtDNA (from a third party), as these genomes have not necessarily co-evolved. Such incompatibilities may conceivably manifest as, for example, dysfunctional protein products or signaling pathways. (**B**) The mixture of two mtDNA types within a cell has been found to cause detrimental physiological effects, for unknown reasons. (**C**) Segregation is the proliferation of one mtDNA haplotype over another in a cellular mixture, potentially causing changes in the population fraction of one mtDNA haplotype. If one mtDNA haplotype experiences a proliferative advantage over another, it may come to dominate the cellular population over time. If some mtDNAs of this haplotype harbor a pathological mutation, this mutation may thus become amplified even if the pathological mutation itself does not affect segregation.

The first potential issue concerns nuclear-mitochondrial interaction (Fig. 3A). Energy production is dependent on extensive cross-talk between genes from the nucleus and mtDNA (Johnson *et al.*, 2001; Reinhardt *et al.*, 2013). Usually the offspring's mtDNA is inherited with a haploid maternal genome. This co-inheritance is thought to facilitate nuclear-mitochondrial interaction. However, during karyoplast transfer this co-transmission is interrupted, and the mtDNA is confronted with a completely ‘unknown’ nuclear DNA. This situation may well lead to complications, as several physiological parameters like respiration, performance ((Nagao *et al.*, 1998), inter-species and/inter-subspecies heteroplasmy) and learning ((Roubertoux *et al.*, 2003), intra-subspecies heteroplasmy) were reduced in mtDNA-nuclear mismatches in male mice. Males are particularly at risk as maternal inheritance of mtDNA implies that the relevant aspects of natural selection only work directly on females, i.e. accumulation of mtDNA mutations that are harmful to males is facilitated, as discussed with LHON. However, recently arguments have been brought forward that male excess in LHON might have other causes (e.g. lower estrogen levels, as estrogen seems to ameliorate mitochondrial dysfunction in LHON (Giordano *et al.*, 2011)), and studies in macaques and mouse models support the view that nuclear-mitochondrial interaction will have limited effect, if at all, on modern treatments (Chinnery *et al.*, 2014). It was recently found that mtDNA haplotypes define gene expression patterns in mouse embryonic stem cells (Kelly *et al.*, 2013), so clearly nuclear DNA (nDNA)–mtDNA interaction does depend on the mtDNA haplotype. However, *in vivo* experiments with xenomitochondrial mice show that the nuclear-mitochondrial system seems to be able to compensate for a high level of diversity. In these xenomitochondrial mice harboring *Mus terricolor* mtDNA on a *Mus musculus* background virtually no negative *in vivo* effects were found (Cannon *et al.*, 2011). In contrast, in conplastic strains that harbored a range of different mtDNAs (*M. m. domesticus*, but also other subspecies of *M. musculus*) and the nucleus of *M. m. domesticus*, behavioral differences and varying susceptibility to experimental autoimmune encephalomyelitis were found (Roubertoux *et al.*, 2003; Yu *et al.*, 2009). Some of these effects might however be caused by a single mutation present in a specific mtDNA haplotype, New Zealand Black (NZB), used in that study (and several others) (Moreno-Loshuertos *et al.*, 2006, 2013). In somatic cell nuclear cloning it was found that a certain mtDNA genetic difference between donor cell and its recipient oocyte can even be beneficial for development. A difference in copy number ratio of mtDNA to mitochondrial mRNA between the respective haplotypes might argue for a necessity for homoplasmy on the mRNA level (Bowles *et al.*, 2008). Thus, while slightly deleterious consequences of nDNA-mtDNA mismatch have been observed in several studies, it seems likely that cells are flexible enough to deal with mismatch situations of limited heteroplasmy and genetic difference.

The second potential issue concerns mtDNA–mtDNA interaction (Fig. 3B). As described above, up to three different mtDNAs can be present in the embryo (excluding low-level microvariation (He *et al.*, 2010; Ye *et al.*, 2014)). Different human mtDNA types show differences in oxidative phosphorylation (OXPHOS), potentially triggered by adaption to various climates or energy demands during evolution, a controversial topic that is still discussed (reviewed in (Wallace and Chalkia, 2013)). What happens if such divergent haplotypes are mixed, even at low levels as after karyoplast transfer? In mice, the mixture of two mtDNA haplotypes of the same subspecies led to physiological changes (e.g. hypertension, changed body mass, blood parameters (Acton *et al.*, 2007)) and altered behavior (Sharpley *et al.*, 2012), while mice carrying 100% of either haplotype stayed healthy. It is likely that low heteroplasmies <5% are insufficient to produce strong manifestations of these effects, but further studies are needed to define the exact heteroplasmy thresholds of importance for mismatching.

The third potential issue concerns mtDNA segregation, that is, the process by which one mtDNA type comes to dominate over another within a cell. The simplest example of this is if mutated mtDNA experiences a proliferative advantage over non-mutated mtDNA, and hence even a small initial amount of mutant mtDNA could eventually come to dominate the cell. As we review below, the evidence to support this positive segregation of mutant mtDNA is scanty, even in diseases where it is known to occur. However, the segregation of non-pathological mtDNAs may be a more pertinent issue in mtDNA therapies. If the mtDNA haplotype of the affected woman (‘donor’) experiences a proliferative advantage over that from the ‘recipient’ healthy oocyte (irrespective of the presence of pathological mutations), an arbitrarily small amount of carryover donor mtDNA could subsequently come to dominate the cellular population (Fig. 3C).

In this scenario, the amplification of donor mtDNA is due to haplotypic differences alone, without any segregation specifically arising from pathological mutations. This mechanism can lead to the amplification of a pathological mutation even if that mutation does not affect segregation. Specifically, if the proliferating donor mtDNA haplotype is associated with a pathological mutation, the amplification resulting from haplotype segregation will lead to a concomitant amplification of the mutation, which ‘hitchhikes’ upon the proliferating haplotype as illustrated in Fig. 3C, potentially reaching pathological levels both in the offspring and subsequent generations.

We focus on segregation effects in particular, for two reasons. First, the aforementioned phenomenon of mtDNA carryover potentially creates a situation in which segregation has to be taken into account: that is, where several different mtDNAs are present within a cell, a situation that has so far rarely been described (St John and Schatten, 2004). Second, as we will subsequently describe, experimental evidence exists to suggest that segregation between different mtDNA haplotypes, though rarely commented upon, may be a significant effect, while evidence regarding the other two issues is more sparse.

### Segregation of pathological mutations

Due to the potentially dramatic physiological implications of mtDNA mutations and progressive segregation documented in tissue culture (Hayashi *et al.*, 1991; Dunbar *et al.*, 1995; Emmerson *et al.*, 2001), one may expect that pathological mutations would lead to extreme segregation effects. While the topic is controversial (Craven *et al.*, 2010), there are good examples of segregation of disease-related mutations in humans (Larsson *et al.*, 1990; Poulton *et al.*, 1995; Weber *et al.*, 1997). To our knowledge, the level of mutant mtDNA is always lower in blood than in post-mitotic tissues such as muscle and brain (Ciafaloni *et al.*, 1992; Larsson *et al.*, 1992; Rahman *et al.*, 2001). A possible cause is the replacement of defective cells (i.e. cells with high levels of mutant mtDNA) with healthy cells in tissues with rapid turnover (Rahman *et al.*, 2001).

In mice containing a mixture of wild-type mtDNA and mtDNA with a 4696-bp deletion (denoted Δ mtDNA) that leads to lethal renal failure, the Δ mtDNA was observed to preferentially accumulate in several tissues over time (e.g. heart, skeletal muscle, kidney, liver, testis and ovary) (Sato *et al.*, 2007). This model, however, is complex and does not clearly recapitulate human disease for three reasons. Firstly, renal failure is uncommon in human mtDNA disease and has rarely, if ever, been reported in mtDNA deletions. Secondly, this rearrangement was maternally inherited, and included mtDNA duplications as well as deletions, both of which are uncommon in human mtDNA disease. Thirdly, the level of mutant mtDNA in the female germline declined with age, a trend that does not reflect the findings in humans (Chinnery *et al.*, 2004). Nevertheless, the model does recapitulate the accumulation of mutant mtDNA in post-mitotic tissues that appears to be the rule in human mtDNA disease.

In a mouse model harboring a slightly deleterious tRNA mutation (m.3875delC), the mutational load was reduced through a mechanism acting at the cellular or organelle level in the developing embryo (Freyer *et al.*, 2012), consistent with observations in inter-subspecies cattle ooplasm transfer described in more detail below (Ferreira *et al.*, 2010). In the mouse model this effect is dependent on the initial heteroplasmy of the mother: offspring from mothers with higher heteroplasmy had lower average heteroplasmy than their mothers. The authors argue that this shift has to take place during gestation, at the cellular or organelle level. However, as no oocytes with >80% of mutated mtDNA could be found, it is possible that this effect (additionally) works on oocyte development.

These findings are consistent with the presence of purifying selection in the germ line, a mechanism acting to eliminate highly deleterious mutations, particularly those located in protein-coding regions. Purifying selection has been directly observed in heteroplasmic mice harboring mtDNA with a severe ND6 mutation along with the wild-type mtDNA. In these mice, the mutation was selectively eliminated during oogenesis within four generations, while a milder cytochrome oxidase 1 (COI) mutation was retained (Fan *et al.*, 2008).

Very recently, in heteroplasmic *Drosophila melanogaster* that harbored a COI mutation that resulted in temperature-sensitive mitochondrial malfunction, it was shown that one possible mechanism of purifying selection is selective propagation of fit mitochondria on the organelle level (Hill *et al.*, 2014).

There is thus some evidence for segregation of pathological mtDNA in animal models, particularly involving selection against mtDNA with demonstrable deleterious effects.

### Segregation of genetically different mtDNA haplotypes

In order to elucidate the mechanisms that govern segregation between non-pathological mtDNA haplotypes, ooplasm transfer and blastomere/cytoplast fusion have been used to create various heteroplasmic animal models using naturally occurring haplotypes that do not specifically harbor a pathological mutation. The best-known example is the heteroplasmic mouse line containing a mixture of NZB mtDNA and a common laboratory mouse strain (CIS) mtDNA (Table I). Laboratory mouse strains show very little variation in mtDNA (Goios *et al.*, 2007), with the NZB strain one of the very few that show considerable genetic difference to the common CIS mtDNA.

**Table I:** Heteroplasmic animal models with physiological mitochondrial DNA (mtDNA) that show *in vivo* segregation and/or physiological changes.

Species	mtDNA/species	Method	Tissue	Physiology	Reference
Mouse	NZB + BALB/cByJ *M. m. domesticus*	Cytoplast fusion	Liver, kidney, blood, spleen	n.a.	Jenuth *et al.* (1997)
Mouse	NZB +129S6 *M. m. domesticus*	Cytoplast fusion	Liver, kidney, spleen, pancreas	Altered behavior	Sharpley *et al.* (2012)
Mouse	NZB + BALB/cByJ *M. m. domesticus*	Cytoplast fusion	n.a.	Altered blood parameters, hypertension	Acton *et al.* (2007)
Mouse	Wild-derived mice + C57/BL6N 3×*M. m. domesticus* 1×*M. m. musculus*	Ooplasm transfer	12 different tissues (various segregation regimes)	n.a.	Burgstaller *et al.* (2014)
Mouse	RR + C57BL/6 *M. m. molossinus/M. m. domesticus*	Blastomere fusion	All; relative to post-mitotic tissue	n.a.	Takeda *et al.* (2000)
Mouse	JF1+ C57BL/6 *M. m. molossinus/M. m. domesticus*	Nuclear transfer	Liver; relative to brain	n.a.	Inoue *et al.* (2004)
Pig	Meishan + Landrace *S. vittatus* *S. scrofa*	Nuclear transfer	Liver; relative to spleen, ear, blood	n.a.	Takeda *et al.* (2006)
Cattle	zebu + taurine cattle *B. p. indicus/B. p. taurus*	Ooplasm transfer	Fetus; (*B. p. indicus* mtDNA reduced during gestation)	n.a	Ferreira *et al.* (2010)

In the NZB/CIS model, the mixture of two naturally occurring but genetically different haplotypes (belonging to the same subspecies) leads to tissue-specific segregation effects: the proportion of NZB mtDNA increases with time in liver and kidney and decreases in blood and spleen. This mixture of mtDNAs leads to detrimental physiological (Acton *et al.*, 2007) and behavioral consequences as described above (Sharpley *et al.*, 2012), although both mtDNAs are regarded free of pathological mutations. Interestingly, the offspring showed a considerable reduction of NZB mtDNA compared with their mothers (Sharpley *et al.*, 2012). The difference was already visible in the oocytes of the mother, but it is likely that the drift also occurs during gestation. This argues for a directed segregation effect operating in the germ line in addition to the aforementioned bottleneck effect.

The basic mechanisms of these segregation effects are largely unknown. One nuclear gene has been found to influence segregation in blood (Gimap3) (Jokinen *et al.*, 2010), and one of the 91 SNPs between the two mtDNA haplotypes was proposed, and hotly discussed, as being responsible via its influence on reactive oxygen species (ROS) production (an ‘A’ track polymorphism in the DHU loop of the tRNA^Arg^ (Moreno-Loshuertos *et al.*, 2006)). Perhaps the most convincing explanation for why heteroplasmy is detrimental is an evolutionary one. Co-evolution of minor differences of *in trans* protein reading frames between divergent haplotypes ensures that multimeric enzyme complexes maintain a high efficiency. However these minor changes will impair efficiency of the complexes when heteroplasmy for divergent haplotypes is present. Of note, the NZB mouse strain generates more ROS than other haplotypes. Even if this ROS production itself is not physiologically deleterious, a deleterious underlying mechanism may be responsible for this difference, driving the tendency of offspring to reduce NZB mtDNA levels – probably on the oocyte and (partly) cellular level (Wallace and Chalkia, 2013).

Ooplasm transfer studies of segregation in other model organisms are limited. In cattle, inter-subspecies ooplasm transfer (*Bos primigenius taurus/B. p. indicus*) has revealed segregation effects during blastocyst development and during gestation, with the *B. p. indicus* mtDNA being removed over time (Ferreira *et al.*, 2010). Also in two inter-subspecific mouse models effects were observed (*M. m. musculus/ M. m. domesticus*, Table I).

However, the NZB model has remained the dominant heteroplasmic model utilizing naturally occurring mtDNA for almost 20 years. A large proportion of our knowledge about mtDNA segregation is based on this most prominent and best-studied heteroplasmic model, yet it is unknown whether its segregational effects represent an exception or a rule, and whether other combinations of mtDNA haplotypes may present different results.

Very recently, to address this question, we produced four mouse models by ooplasm transfer, placing various naturally occurring mtDNA haplotypes from mice captured from the wild in Europe onto a common laboratory mouse mtDNA and nuclear background (C57BL/6N). The wild-derived haplotypes we used display a spectrum of genetic differences with C57BL/6N, thus enabling us to control genetic distance (from very similar haplotypes, to haplotypes that differ in a comparable number of sites to two randomly chosen human mtDNAs, as described above). We also developed a mathematical framework to facilitate the direct comparison of many of these mice. We found that tissue-specific segregation was very common (including within post-mitotic tissue types), with the magnitude of segregation increasing with the genetic distance between the mtDNA haplotypes, and identified several contrasting mechanisms related to mtDNA turnover and organismal age by which this segregation occurred (Burgstaller *et al.*, 2014). This study suggests that segregation between naturally occurring haplotypes may be the rule rather than exception, particularly with genetically diverse mtDNA pairings.

Heteroplasmy also exists, and has been studied, after nuclear transfer; but most studies have investigated the effects of the transfer process itself rather than focusing on mtDNA heteroplasmy. Nevertheless, these studies cover several species (cattle, sheep, pig, mouse), and cover inter- and intra-species heteroplasmy (reviewed in detail in St John *et al.* (2010)), and provide a body of data demonstrating co-existence of two mtDNA haplotypes in several species *in vivo*. Several studies report aberrance from expected donor mtDNA amounts that could be caused by segregation bias (Hiendleder *et al.*, 1999; Takeda *et al.*, 2003; Burgstaller *et al.*, 2007). In particular, one study systematically analyzing cloned pigs and their offspring demonstrated powerful segregation effects between mtDNA from the genetically distant Meishan and Landrace breeds, which represent two subspecies of *Sus scrofa*. In these animals, the Meishan mtDNA significantly increased in liver, relative to spleen, ear and blood ((Takeda *et al.*, 2006), Table I). Another inter-species study in mouse (*M. m. molossinus*/*M. m. domesticus*), also found segregation in liver, with the *M. m. molossinus mtDNA* increasing (measured relative to brain (Inoue *et al.*, 2004)).

It is notable that in studies observing many animals over a substantial amount of time, or over several generations, segregation between different mtDNA types is often observed (Table I). Interestingly, in all studies of post-natal animals, liver is the tissue with the highest segregation effect. We can only speculate why this might be, but note that liver tissue has a high energy demand combined with high mtDNA turnover. Liver mtDNA half-lives are estimated at between 2 (Miwa *et al.*, 2008, 2010) and 9 days (Gross *et al.*, 1969; Menzies and Gold, 1971; Korr *et al.*, 1998) when compared with, for example, skeletal muscle (reports from 18 (Korr *et al.*, 1998) to 700 days (Collins *et al.*, 2003)). The fast turnover time of mtDNA in liver and potentially strong selective pressure for energy production may underlie the rapid segregation observed in this tissue.

## Implications

We have reviewed classical and modern approaches to address the inheritance of mtDNA disease. Modern approaches—pronuclear transfer and spindle transfer—have the potential to ameliorate mtDNA disease without the unsatisfactory genetic features of classical approaches. However, we have noted that several uncertainties are currently associated with the post-therapy behavior of embryos created using these techniques. These issues include mtDNA-mtDNA and mtDNA-nDNA mismatches, which could be important at high heteroplasmies, but are likely dampened by the ability of modern therapies to guarantee <1–2% donor carryover. Segregation of pathological mtDNA is potentially damaging. A key argument for nuclear transfer is therefore that current evidence suggests such segregation is either of very low magnitude or acts in such a way to remove pathological mutation (or both), and hence is likely not a key issue in mtDNA therapies (Craven *et al.*, 2010). While further work is required to satisfactorily characterize these phenomena, the evidence suggests that they might not pose immediate issues in the application of genetic therapies.

The remaining phenomenon, segregation of non-pathological mtDNA haplotypes, is possibly the most important unaddressed question associated with modern mtDNA therapy, due to the potential consequent amplification of pathological mutations associated with one haplotype in the offspring resulting from genetic therapy, and in subsequent generations.

A key clinical consideration is whether segregation, and subsequent potential amplification of pathological mutations, could occur in post-mitotic tissues in which mtDNA diseases are most often manifest (Reeve *et al.*, 2008). In organs where cells are constantly renewed (for example, skin and intestine), cells with damaged OXPHOS systems are probably replaced by functioning ones (Rahman *et al.*, 2001). Organs particularly at risk for mtDNA disease are the post-mitotic tissues of heart, and skeletal muscle and brain; and liver and kidney which show high (liver) (Michalopoulos and DeFrances, 1997) and rather limited (kidney) (Little, 2006) regenerative potential. Our recent study using wild-derived mouse mtDNA has demonstrated haplotypic segregation in heart and skeletal muscle (Burgstaller *et al.*, 2014). Liver and kidney are both tissues that show mtDNA segregation bias in the famous heteroplasmic mouse model described by the Shoubridge group described above (Jenuth *et al.*, 1997).

An important question when considering the implications of animal models is whether phenomena observed in animals also occur in humans. Investigation of mtDNA dynamics during human development is practically limited for clear reasons, and the mouse NZB model remains by far the best-studied model of mtDNA haplotype segregation. However, reports of coexistence between two mtDNA haplotypes, and reports of mtDNA segregation effects, are present across several species, suggesting that these effects may be shared by all mammals. Further studies are however needed to confirm this assumption, and, importantly, elucidate the mechanisms on which these effects are based.

Based on the current available evidence, we believe that segregation between different naturally occurring mtDNA haplotypes may potentially influence the post-therapy behavior of intracellular mtDNA populations in offspring produced through modern gene therapies. To recap, these therapies involve recruiting a ‘recipient’ oocyte to serve as a healthy mitochondrial background for nuclear DNA resulting from fertilization. However, experimental limitations mean that some of the original mother's ‘donor’ mtDNA will inevitably be present in embryos produced in this way. If donor mtDNA proliferates over recipient mtDNA, the donor mtDNA will become amplified during development and during the lifetime of the offspring. If the donor mtDNA is associated with a pathological mutation, even if this mutation does not affect mtDNA proliferation, its ‘hitchhiking’ on the proliferating mtDNA may cause its amplification to potentially pathological levels in the offspring. We note that this worst-case haplotype segregation will not, in itself, cause additional harm to offspring beyond that expected from mtDNA disease inheritance; rather, it has the potential to nullify the beneficial effects of genetic therapy by re-establishing the original mtDNA mixture that was present in the donor oocyte, possibly in a tissue-specific way. The possibility also exists that the amplification of one mtDNA type through segregation may affect the behavior resulting from the aforementioned mtDNA-mtDNA mismatch, which is very likely suppressed at low heteroplasmies.

It is notable that all potential mtDNA segregation issues (indeed, all three of the potential issues we note) associated with modern mtDNA therapies can be ameliorated by employing a simple ‘haplotype matching’ protocol: that is, ensuring that the donor and recipient mtDNA haplotypes are as similar as possible. This approach will minimize nDNA-mtDNA mismatching (as the donor nucleus will have co-evolved with donor mtDNA, very similar to recipient mtDNA); mtDNA-mtDNA mismatch (due to the genetic similarity); and mtDNA segregation (as two very similar haplotypes are expected to show very little segregation). The ideal recipient would be of the same haplotype as the donor (minus the pathological mutation), for example, from a healthy maternal relative. Alternatively (or in addition), further research on the segregation of different pairs of mtDNA haplotypes could be used to choose suitable recipients for a given donor, in order to minimize segregation effects.

Experts in the field of karyoplast transfer have noted that ‘it is possible to match mitochondrial haplotype between the mother and the mitochondrial donor to avoid any concern, even though the evidence says it should not be needed’ (Chinnery *et al.*, 2014). We think that the somewhat overlooked issue of mtDNA segregation currently constitutes a reason that merits this safety precaution, which would solve all potential concerns reviewed here. Additionally, in the case of exact haplotype matching, offspring mtDNA would have a complete genetic identity with the mother's mtDNA, possibly going some way towards alleviating the ethical issues associated with ‘three-parent babies’; that is, offspring with genetic material from mother, father and a third party.

While uncertainties exist regarding the behavior of mixed intracellular mtDNA populations, and animal models of mtDNA mixtures during development suggest that segregation potentially requires further study and consideration in therapeutic contexts, it should firmly be noted that these recent mtDNA-replacement strategies hold the promise to eliminate transmission of mtDNA diseases for good, and in so doing dramatically improve the lives of families carrying mtDNA disease. The potential advantages of these therapies seem to, in general, substantially outweigh their known risks. The unknown risks must thus be balanced against the certainties of classical genetic management. Hence potential patients for the first treatment trials will be from the rare homoplasmic families at proven high recurrence risk of severe phenotypes, for whom classical genetic management has least to offer. Initiating clinical trials is the only way to evaluate the presently unknown risks and future hopes for families brought by modern mtDNA therapies.

## Authors' roles

J.P.B and I.G.J. compiled the content and produced the manuscript; J.P. edited the manuscript and provided clinical input.

## Funding

I.G.J. gratefully acknowledges funding from the UK Medical Research Council; J.P. is supported by the Medical Research Council (MR/J010448/1) and the Wellcome Trust (WT0948685MA). Funding to pay the Open Access publication charges for this article was provided by the Medical Research Council.

## Conflict of interest

None declared.
